# RSV Infection and Neurodegenerative Diseases: A Hypothesis of Energy Metabolism Disruption *via* the Lung-Brain Axis

**DOI:** 10.14336/AD.2025.0534

**Published:** 2026-05-13

**Authors:** Zeping Liu, Yan Zhou, Mingming Qi, Fangyuan Ren, Yurong Tan

**Affiliations:** ^1^Department of Obstetrics, Zhuzhou Hospital Affiliated to Xiangya School of Medicine, Central South University, Changsha, China.; ^2^Department of Medical Microbiology, Central South University Changsha, Hunan, China.

**Keywords:** Lung-brain axis, RSV, Neurodegenerative diseases, Energy metabolism

## Abstract

Respiratory Syncytial Virus (RSV), primarily recognized as a respiratory pathogen, has emerged as a potential contributor to neurodegenerative diseases via the "lung-brain axis." Preclinical studies highlight RSV-induced energy metabolism dysfunction as a core pathological mechanism encompassing mitochondrial dysfunction, glucose metabolism reprogramming, and microglial metabolic polarization-critical gaps in clinical validation, molecular specificity, and translational relevance. This review addresses these limitations by advocating for enhanced epidemiological research, detailed molecular pathway characterization, and the integration of human-relevant models. Targeted metabolic interventions have been proposed, supported by recent mechanistic insights, to bridge the gap between hypotheses and therapeutic development.

## 1. Introduction

Respiratory syncytial virus (RSV) belongs to the genus Paramyxoviruses of the family Paramyxoviridae. It is an enveloped virus with a negative-stranded RNA genome and a spike protein (F and G proteins) on its surface, which is used for binding and fusion with host cells [1]. RSV is a highly contagious pathogen associated with a high burden of lower respiratory tract infections, particularly in the elderly and infants under 2 years of [2-5]. For most healthy adults and children, RSV typically causes mild cold symptoms; however, for high-risk populations, it may lead to severe respiratory diseases and require hospitalization, which puts great pressure on socio-economic and medical systems [6-8].

1-7% of children hospitalized due to RSV infection may experience certain neurological complications, including encephalitis, complex epileptic seizures, and status epilepticus [9,10]. There is ample evidence to suggest that RSV can directly infect cells within the central nervous system (CNS) [11,12], and that the significant inflammatory response caused by RSV infection in the respiratory tract may affect the CNS and may be associated with the occurrence and development of neurodegenerative diseases [13,14]. These findings have opened up new directions for the study of RSV, while also sparking extensive discussion on its potential neurotoxic mechanisms.

Neurodegenerative diseases, such as Alzheimer's disease (AD) and PD, are often closely associated with protein homeostasis imbalance, mitochondrial dysfunction, and abnormal energy metabolism. Although inflammation and direct nerve damage are considered to be the main mechanisms by which viral infections cause neurodegeneration, increasing evidence suggests that energy metabolism disorders may play a key role. RSV infection induces host cells to produce large amounts of reactive oxygen species (ROS), leading to impaired mitochondrial function and reduced ATP production, which in turn affects the survival and function of neurons and microglia. In addition, RSV may further exacerbate the energy crisis in nerve cells by altering glucose metabolism pathways.

Based on the above background, this article proposes the “energy metabolism disorder hypothesis”, aiming to explore how RSV infection interferes with the energy metabolism of nerve cells, ultimately leading to neurodegeneration. By systematically reviewing the neural invasion mechanism, metabolic abnormalities, and their association with neurodegenerative diseases of RSV, this article provides a new perspective for understanding the neurotoxicity of RSV and lays a theoretical foundation for developing therapeutic strategies targeting metabolic pathways.

## 2. RSV and neurodegenerative diseases: the pathway from the lungs to the CNS

### 2.1 The concept of the lung-brain axis

Chronic or severe respiratory infections can induce neuroinflammation, which is linked to neurodegenerative diseases such as AD, PD, multiple sclerosis, and autism. Research has found that after injecting retrograde-tracing viruses into lung tissue, corresponding fluorescent expression was observed in multiple brain regions, indicating a close anatomical connection between the lung and brain [15]. A recent study published in Nature revealed that lung microorganisms play a crucial role in CNS immunity by regulating the immune responsiveness of central nervous tissue, thereby influencing its susceptibility to autoimmune disease development [16]. Additionally, a clinical study discovered a relationship between the disease progression of pulmonary arterial hypertension and changes in neurocognitive function. They suggested that pulmonary arterial hypertension patients with significant cognitive impairment have a higher risk and poorer prognosis than those without [17]. These findings validate the existence and function of the lung-brain axis [18]. This presents a novel perspective suggesting that respiratory infections may be a significant risk factor for neurodegeneration. Viruses may also affect brain function through peripheral infection; the blood-brain barrier; and immune, metabolic, and neural regulation.

Research by Kariem et al. indicates that RSV infection may uphold the pathological changes associated with AD, potentially facilitating aggregation of amyloid proteins, which detrimentally impacts neurons and impairs cognitive function [19]. Additionally, RSV-induced immune reactions may trigger toxic effects on neurons, fostering further progression of this condition. The influence of RSV on both neural damage and cell death is also evident in PD. RSV infections can induce neuronal injury via apoptosis induction and the subsequent self-destruction of infected cells through intrinsic signaling pathways. Studies have suggested that viral infections may precipitate or intensify multiple sclerosis episodes. Although research remains ongoing, evidence suggests that maternal RSV infection during pregnancy may increase offspring risk for autism [20].

### 2.2 Ways RSV invades the nervous system

**Figure 1.**
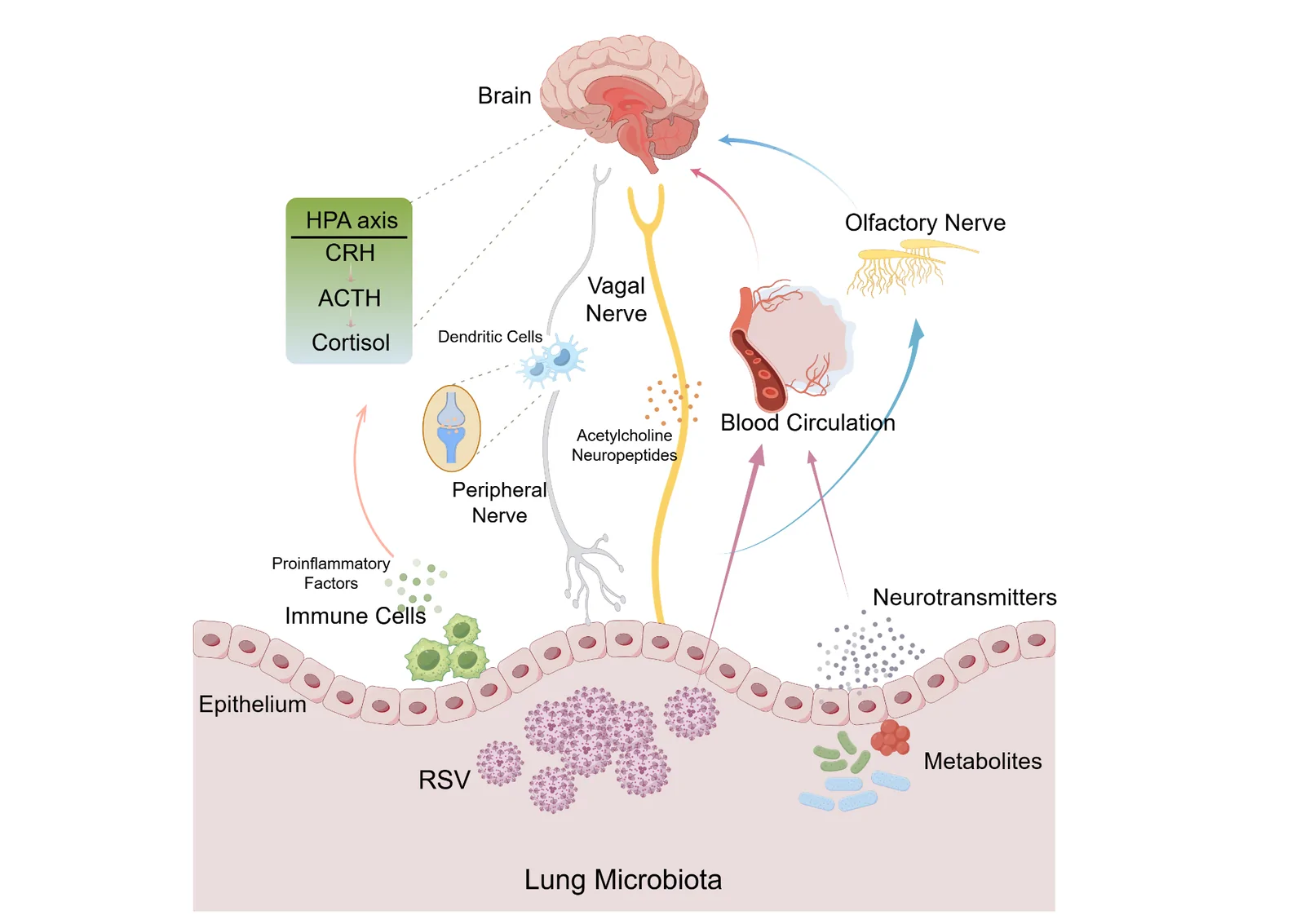
Possible lung-brain pathway for RSV-exacerbated neuropathy.

At present, there is relatively little research on the mechanisms related to the lung-brain axis. The known or predicted potential mechanisms include direct translocation, vagus nerve conduction, cytokine regulation, neuroendocrine signaling, transmission through the peripheral nervous system and microbial metabolite diffusion (Fig. 1). RSV is the main pathogen that causes respiratory diseases, and its infection in the lungs can lead to remodeling of the nervous system [21]. Increasing evidence suggests that RSV is not limited to the respiratory tract but can also spread to non-respiratory tissues, potentially invading the nervous system and disrupting its normal function [22].

#### 2.2.1 Direct Translocation

Research has indicated that up to 90% of respiratory infections are caused by viruses. Strikingly, these respiratory viruses can penetrate olfactory sensory neurons, infecting their axons projecting to the olfactory bulb, thereby circumventing the blood-brain barrier via the olfactory pathway into the CNS [23-25]. Traditionally, RSV is believed to enter the CNS via blood circulation, but studies suggest that it can also interact with the lungs and brain through neural pathways [26]. RSV mRNA has been detected in CSF samples from patients with altered neurological function, indicating its potential entry into the CNS [27,28]. Mao et al. observed an increase in mRNA expression levels in the brains of RSV-infected mouse models using IFA [29].

One possible mechanism of RSV invasion is olfactory nerve anterograde transport [30]. This pathway is advantageous for viruses entering the CNS from the nose because the olfactory nerve has unique connections with the nasal epithelium and olfactory bulb [31,32]. Studies have confirmed RSV's ability of RSV to invade the nervous system via the olfactory route by in-nose injection of virus and observing the viral genome and protein in the infected mouse brain region [33]. Another potential entry route could be the hematogenous route. Following primary infection, RSV, a neurotropic virus, can access the bloodstream and reach the CNS, a process known as viremia [34]. RSV employs multiple viral proteins to facilitate neuroinvasion and disrupt host metabolism. For example, non-structural protein 1 (NS1) induces mitochondrial autophagy by promoting mitochondrial-lysosome fusion in HEp-2 cells, a process linked to ATP depletion and ROS accumulation [35]. The SH protein interacts with the host cytoskeletal protein KRT9, destabilizing mitochondrial-endoplasmic reticulum junctions and impairing calcium homeostasis, which are key steps in mitochondrial dysfunction [36]. These interactions highlight RSV’s specificity of RSV in hijacking mitochondrial quality control pathways, a mechanism distinct from other respiratory viruses such as influenza, which primarily disrupt mitochondrial dynamics via M2 protein-mediated ROS induction [37].

#### 2.2.2 Vagal Nerve Transmission

The vagus nerve plays a crucial role in the lung-brain axis mechanism. It mediates communication between the lungs and brain via its intricate transmission function. Its sensory neurons, the primary source of neural fibers innervating the lungs and airways, sense changes in airflow, gas composition, and lung tension, transmitting this information to the respiratory center of the brain via sensory neurons, thereby regulating the respiratory frequency and depth [38]. The vagal sensory neuron tips, central integration center, acetylcholine and a7 nicotinic acetylcholine receptor-expressing cells collectively form the key components of the pulmonary parasympathetic nerve inflammation reflex. Upon stimulation, they can regulate the functional state of the lungs and brain by releasing acetylcholine or neuropeptides [39]. Moreover, vagus nerve function is closely linked to psychological and emotional states, potentially influencing mood and cognitive conditions. For example, studies indicate that peripherally delivered mesenchymal stromal cells can activate vagal sensory neurons within the lungs, further inducing the release of serotonin from the dorsal raphe nucleus, ultimately improving aberrant behavior patterns in depressed mice [40]. Additionally, research has found that IL-1β can activate airway C fiber receptors and high-threshold δ opioid receptors, which may influence innate immune responses by activating the CNS.

#### 2.2.3 Cytokine Regulation

Cytokines, molecules secreted from cells that regulate immune responses, inflammation, cell proliferation, and differentiation, include interferons, chemokines, interleukins, and tumor necrosis factors. Pulmonary microorganisms stimulate pro-inflammatory mediators that affect systemic humoral factors, triggering extensive inflammation. Cytokine regulation in the lung-brain axis mechanism involves bidirectional signal transmission between the lungs and brain and is significant in chronic diseases such as chronic obstructive pulmonary disease, asthma, and depression. Understanding this mechanism is crucial for improving the treatment of chronic lung diseases and their related neurological symptoms.

The encephalitis caused by RSV can be classified into four categories: (1) metabolic error, (2) cytokine storm, (3) excitotoxicity, and (4) hypoxic encephalopathy [41]. The second classification denotes a significant systemic elevation of several cytokines. RSV mobilizes cytokines and chemokine receptors to aberrantly alter neuronal functionality during systemic inflammation induction [42,43]. Previously published research detected elevated expression of the pro-inflammatory cytokines IL-6 and IL-1β during viral infection [29]. Secreted pro-inflammatory cytokines exacerbate neuropathic inflammation, culminating in synaptic degeneration and neuronal cell death, with a robust correlation between cytokine imbalance and neurological disorders, including neuroinflammation-related depression [44]. Additionally, studies have found that pulmonary microorganisms influence the Th1 immune response induced by RSV infection, ultimately inducing a microglial phenotype shift in mouse brain tissues [45]. Microglia, which are the resident immune cells of the brain, actively regulate the extracellular environment of both healthy and disordered brains [46]. These cells are extremely sensitive to inflammation activation or trauma from diverse inflammatory stimuli and, once activated, secrete various pro-inflammatory molecules, such as IL-1, TNF-α, and IL-6, which critically disrupt the brain's higher functions [47,48]. Consequently, this finding suggests that the augmented expression of these pro-inflammatory cytokines might be caused by microglial activation. Thus, airway RSV infection via the lung-brain axis potentially triggers neuroinflammation, initiating neurodegeneration, and behavioral alterations.

#### 2.2.4 Neuroendocrine Signal Transduction

The neuroendocrine system is a biological network with both neural and endocrine functions, tasked with regulating internal body equilibrium. Neuroendocrine signal transduction refers to the physiological and pathological processes prompted by signals passing cascades of substances, such as neurons and neurotransmitter receptors. The hypothalamohypophysial -adrenal (HPA) axis serves as the core of the neuroendocrine system and plays a pivotal role in stress response, immune regulation, neurological function, and homeostatic maintenance. The hypothalamic-pituitary-adrenal (HPA) axis has been extensively studied in experimental and clinical settings and is potentially linked to lung dysfunction. Systemic glucocorticoids are primarily synthesized by the adrenal cortex under the control of the HPA axis. Research indicates that the lungs of mice possess steroidogenic enzymes necessary for glucocorticoid synthesis, suggesting that locally produced glucocorticoids may influence the local environment, cell development, and immune cell activation via paracrine or autocrine mechanisms [49,50]. Glucocorticoids are widely present in the CNS and are capable of binding to glucocorticoid receptors, influencing inflammatory factors and microglial activation, thereby playing a crucial role in central inflammation. A multicenter randomized trial reported 150 intubated patients with severe trauma and insufficient cortisol, where stress dose hydrocortisone reduced the risk of acquired pneumonia, particularly in a subgroup of patients with severe traumatic brain injury [51]. As the primary stress response system, HPA axis activation may alter the lung microbiome, warranting further exploration of its potential role in the lung-brain axis.

#### 2.2.5 Transmission through the peripheral nervous system

RSV may also exploit the peripheral pulmonary nerve innervation as a conduit to enhance access to the CNS.

RSV has been shown to infect neurons inefficiently, including those innervating the lungs of mice, and can directly infiltrate the peripheral autonomic and nociceptive nerve fibers in the airways [52-54]. Subsequently, the virus can travel along peripheral nerve fibers and be released within the CNS during subsequent synaptic transmission or neuronal death [31,55]. Research indicates that in peripheral nerves and spinal cord tissue, RSV-infected dendritic cells persist longer than macrophages, suggesting that dendritic cells may serve as long-term reservoirs for RSV in the peripheral nervous system and CNS [56].

#### 2.2.6 Metabolite Pathways

RSV infection alters metabolic activity within the lungs and brain [45,57]. This intricate process involves not only host-derived metabolites, but also a plethora of metabolites produced by the body's microflora, including neuroprotective or damaging metabolites that regulate immune responses and cerebral function [58,59]. Despite its importance, the precise mechanism by which RSV infection modulates the host microbiota and its subsequent influence on CNS signaling remains unclear, posing significant challenges in devising therapeutic strategies and sparking intense international research interest.

## 3. RSV triggers neurodegenerative diseases: hypothesis of energy metabolism disorders

**Figure 2.**
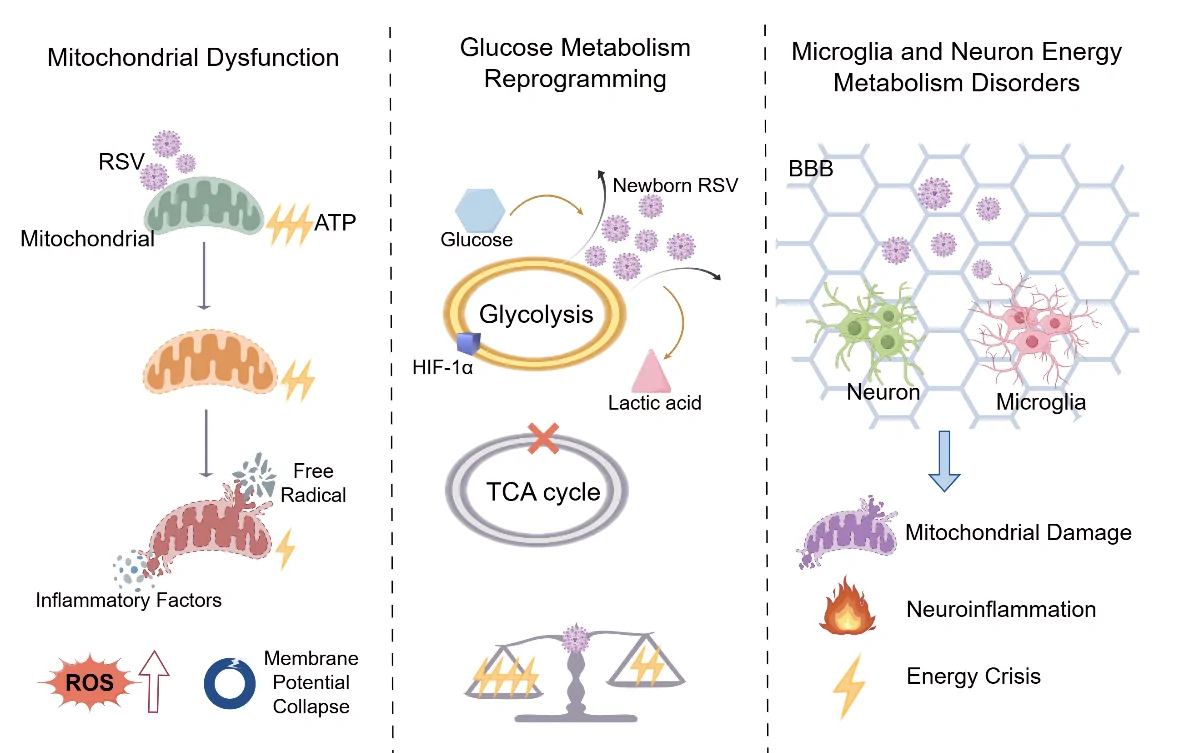
Possible pathways of energy metabolism caused by RSV infection.

Energy metabolism is the core foundation of cellular physiological functions including ATP generation, distribution, and utilization. The nervous system is particularly dependent on energy requirements, especially neurons and microglia, which require a large amount of energy to maintain their basic functions, such as nerve conduction, synaptic plasticity, and immune response. RSV infection may interfere with this system via several pathways, leading to impaired energy metabolism. As illustrated in Figure 2, there are three possible approaches that are described in detail.

### 3.1 mitochondrial dysfunction

RSV infection may affect homeostasis of the nervous system by interfering with the energy metabolism of host cells, particularly mitochondrial function. Mitochondria, as the energy factory, play a crucial role in maintaining the energy supply, regulating redox balance, and mediating cell apoptosis. RSV infection leads to mitochondrial dysfunction through various mechanisms, such as oxidative stress, membrane potential collapse, morphological changes, and host defense mechanism failure, ultimately causing energy metabolism disorders and accelerating neurodegeneration. In addition, RSV infection not only activates the immune response but may also further impair mitochondrial function by increasing oxidative stress and releasing cytokines. Owing to the high demand for ATP in nerve cells, mitochondrial dysfunction may lead to insufficient energy in neurons and microglia, thereby affecting their function and health.

In the long-term evolutionary process, viruses have developed multiple mechanisms to promote their survival and replication by interfering with mitochondrial metabolism. These mechanisms include inhibiting mitochondrial oxidative phosphorylation, damaging mitochondrial DNA (mtDNA), interfering with signal protein function, and altering mitochondrial generation and degradation, ultimately leading to mitochondrial dysfunction. For example, HCV, dengue virus (DENV), Zika virus, and human immunodeficiency virus (HIV) can reshape host cell metabolism, enhance mitochondrial fatty acid oxidation, and provide energy support for virus replication [60-62]. HCV cleaves MAVS protein through NS3/4A protease, inhibiting PINK1/Parkin mediated mitochondrial autophagy, leading to accumulation of HIV inhibits mitochondrial autophagy by suppressing PINK1 expression, leading to neuronal dysfunction. Similarly, influenza virus induces ROS accumulation through the M2 protein, inhibits the PINK1/Parkin pathway, and leads to dysregulation of mitochondrial autophagy.

Hu et al. showed that staged redistribution of host cell mitochondria in RSV-infected cells leads to impaired respiratory activity and increased ROS generation, resulting in mitochondrial membrane potential collapse and reduced ATP production. These results highlight the unique ability of RSV to seize host cell mitochondrial ROS and promote viral infection [63]. Simultaneously, the SH protein of RSV interacts with the host cell cytoskeleton protein KRT9, disrupting the mitochondrial endoplasmic reticulum junction, interfering with calcium ion homeostasis, and exacerbating mitochondrial dysfunction [64]. The epidermal growth factor receptor (EGFR) protein is a 170 kDa transmembrane receptor that is important for maintaining mitochondrial homeostasis and EGFR activity in human cell growth. The translocation of EGFR into the mitochondria affects the production of ATP and ROS, which contribute to mitochondrial bioenergetics. The entire EGFR signaling pathway network is crucial for maintaining cellular homeostasis, controlling cell survival, and maintaining balance [65,66]. Research has shown that in the early stages of RSV infection, the activation of EGFR, maintenance of mitochondrial function, and ATP levels promote cell survival and viral replication [64]. Another study suggested that RSV induces mitochondrial autophagy through the ROS-AMPK signaling axis, thereby promoting viral infection. Autophagy promotes RSV replication by blocking apoptosis at 48 hpi. Mechanistically, RSV induces mitochondrial autophagy and maintains mitochondrial homeostasis, thereby reducing the release of cytochrome c and inducing apoptosis [64]. RSV-induced mitochondrial damage is mediated by both direct viral-protein interactions and host immune responses. The NS1 protein, for instance, disrupts the PINK1/Parkin-mediated mitophagy pathway, leading to the accumulation of dysfunctional mitochondria with swollen cristae and reduced ATP synthase (ATP5A) expression [35]. Concurrently, the SH protein impairs EGFR-mediated mitochondrial homeostasis, destabilizes ATP production, and increases ROS levels [64]. These mechanisms converge to force neurons into an inefficient glycolytic state, a metabolic switch exacerbated by HIF-1α stabilization driven by both viral-induced ROS and mitochondrial membrane potential collapse [65].

Although RSV infection has been shown to impair mitochondrial function in neurons, its impact on long-term neuronal survival in humans remains unclear, particularly given the compensatory mechanisms observed in aged primate models.

### 3.2 Glucose metabolism reprogramming

RSV infection activates the immune response in the brain through the action of the lung-brain axis, including the activation of microglia. Research has shown that immune cells undergo metabolic reprogramming when exposed to viral infections, which is typically manifested as a shift from oxidative phosphorylation to glycolysis pathways to respond to the demands of acute immune responses. However, metabolic reprogramming may lead to an unstable energy supply, resulting in a cellular energy crisis. In addition, under chronic inflammatory conditions, this metabolic change can also affect neuronal function, ultimately leading to symptoms such as cognitive impairment and neurodegeneration. Glycolysis is a series of oxidative reactions used to metabolize glucose and provide energy to host cells. It is also necessary for RSV infection and supporting self-replication. Previous studies have found that RSV infection promotes glycolysis in primary human alveolar epithelial cells and bone marrow-derived cells [66,67].

RSV activates the insulin receptor (IR)-PI3K-Akt axis, upregulating HIF-1α, which in turn induces glucose transporters (Glut1, Glut3) and glycolytic enzymes (HK1 and PFKP) [67]. While this enhances glycolytic ATP production (Glyco ATP), it suppresses oxidative phosphorylation, as evidenced by reduced mitochondrial ATP (mito ATP) and disrupted tricarboxylic acid (TCA) cycle flux [68]. Downstream, lactate accumulation from glycolysis exacerbates neuronal damage by promoting microglial M1 polarization and ROS-dependent synaptic dysfunction [69]. Single-cell RNA sequencing studies have revealed that neuronal subpopulations (e.g., hippocampal neurons) are particularly vulnerable to this metabolic reprogramming, highlighting cell-type specificity in RSV-induced energy crises. In addition, the generation of ROS further stabilizes and activates HIF-1α, forming a positive feedback loop and forcing host cells to shift from an energy supply mode dependent on mitochondrial oxidative phosphorylation (OXPHOS) to a metabolic mode dominated by glycolysis. Energy spectrum analysis showed a significant increase in ATP produced by glycolysis after infection, whereas the rate of mitochondrial ATP production decreased, confirming this metabolic reprogramming phenomenon.

At the mechanistic level, the metabolic imbalance caused by RSV can be attributed to the virus's "hijacking" of host energy resources. HIF-1α- mediated glycolysis not only provides energy for virus replication but also directly supports the assembly and release of progeny virus particles. RSV satisfies its own energy needs by forcing host cells to enhance glycolysis, ultimately leading to the breakdown of energy metabolic homeostasis.

RSV infection of epithelial cells in vitro leads to increased central metabolism and overall metabolic hyperactivity; however, little is known about whether natural RSV infection in vivo leads to persistent metabolic reprogramming of the airway epithelium in infancy. To address this gap, Connelly et al. performed functional metabolomics, 13C glucose metabolism flux analysis, and RNA-seq gene expression analysis on nasal airway epithelial cells (NAECs) sampled from 2-3 year old infants, regardless of RSV infection in the first year of life [70]. They found that RSV infection in infancy is associated with sustained epithelial metabolic reprogramming, characterized by (1) significantly increased glucose uptake and differential utilization of glucose by the epithelium; (2) altered metabolic preferences for several carbon and energy sources; and (3) significant sexual dimorphism in metabolic parameters, with RSV-induced metabolic changes being most pronounced in male epithelium. This research supports the proposed phenomenon of epithelial cell metabolic reprogramming associated with RSV infection in infancy and expands the scope of studying the mechanisms of RSV-induced early epithelial barrier dysfunction.

### 3.3 Energy metabolism disorders of microglia and neurons: the core mechanism of RSV exacerbated neurodegeneration

The energy metabolism disorders caused by RSV infection are not limited to the peripheral tissues. After invading the CNS through the blood-brain barrier or neural pathways, they can directly or indirectly interfere with the energy metabolism homeostasis of the microglia and nerve cells. This metabolic disorder may be the core mechanism driving neurodegeneration through a vicious cycle of “mitochondrial damage and energy crisis resulting from neuroinflammation”. As immune cells of the nervous system, microglia require a large amount of energy to carry out immune responses during infection, including cell migration, cytokine secretion, and intracellular clearance. Excessive energy expenditure may lead to microglial dysfunction, further affecting the supportive function of neurons and the regulation of neuroinflammatory responses. Microglia are immune cells in the brain that participate in immune monitoring and the response of the nervous system. During RSV infection, microglia are activated to clear the virus and its pathological products; however, this process is accompanied by significant energy expenditure. Research has shown that, during viral infection, the energy demand of microglia increases sharply, especially when they transition from a "dormant" state to an "active" state. Microglia typically rely on the mitochondrial energy supply to cope with infections while also increasing ATP production through the glycolytic pathway. However, overactivation of microglia may lead to the following problems:

1. Insufficient energy supply: Activated microglia require a large amount of energy for phagocytosis, cytokine secretion, and the maintenance of their high metabolic state. If the energy metabolism system is inhibited (such as mitochondrial dysfunction and dysregulation of glycolytic pathways), microglia will be unable to efficiently execute immune responses, leading to weakened neuroprotective ability.
2. Energy imbalance caused by excessive inflammation: Persistent neuroinflammatory reactions not only increase the metabolic burden on microglia but may also cause energy metabolism disorders in nerve cells, leading to functional damage and death of nerve cells. Overactivation of microglia, especially in the absence of sufficient energy support, may lead to the release of excessive cytokines, such as TNF-α and IL-1β, thereby promoting energy metabolism disorders and damage to neurons.

A study based on three-dimensional microphysiological peripheral nerve culture revealed two different trajectories of RSV infection in the nervous system depending on the initial viral load [71]. In cases of low viral load, the infection exhibits transient characteristics, mainly affecting macrophages and triggering moderate levels of chemokine secretion, accompanied by short-term neural hypersensitivity. In contrast, when the viral load is high, the infection becomes persistent, not only affecting monocytes but also directly invading neurons, triggering the release of a large number of chemokines, which in turn leads to progressive neurotoxicity. In addition, in spinal cord culture experiments, RSV preferentially infects microglia and dendritic cells, with less direct infection of neurons and moderate expression of chemokines. It is worth noting that infections in dendritic cells can last for up to 30 days. These results indicate that RSV can indirectly disrupt neuronal function by directly infecting surrounding neurons or by infecting resident monocytes, such as microglia and dendritic cells, which may play a key role in both mechanisms.

The effect of RSV on microglia is mainly mediated by the induction of their activation and pro-inflammatory phenotype polarization, leading to neuronal damage and CNS dysfunction. Although the specific receptors for RSV infection of microglia have not been fully elucidated, previous studies have shown that RSV may mediate infection through receptors, such as CX3CR1, nucleolin, EGFR, IGF1R, HSPG, and ICAM-1 [72]. After infection, the expression of the small glial cell marker Iba-1 increases, while the neuronal marker NeuN decreases, suggesting that RSV infection is associated with microglial activation and neuronal damage [29]. Activated microglia switch from oxidative phosphorylation to glycolysis during RSV infection, a shift marked by increased expression of voltage-gated proton channels and pro-inflammatory markers (IL-1β and iNOS) [73,74]. This "M1 polarization" releases cytokines (TNF-α, IL-6) and ROS, creating a vicious cycle: neuronal mitochondrial damage elevates ROS, which further activates microglia, while glycolytic metabolites (e.g., lactate) disrupt synaptic plasticity [45]. These mechanisms may explain the association between RSV infection and long-term neurological sequelae such as seizures and encephalitis [29,45,72].

### 3.4 Gap between experimental models and clinical reality: limitations of RSV neuropathological research

Although existing in vitro and mouse studies have suggested that RSV infection may trigger neurodegeneration through metabolic dysfunction, their limitations require careful consideration. While in vitro models can directly assess RSV's effects of RSV on mitochondrial function and glycolysis, they lack the complex cellular interactions and systemic metabolic regulation (e.g., hormonal signaling and inter-organ crosstalk) present in vivo. For instance, the observed glycolytic shift in cultured cells may be exaggerated by high-glucose media, whereas neurons in vivo primarily rely on oxidative phosphorylation, suggesting a higher threshold for metabolic disruption [73].

While murine models have demonstrated that RSV infection can induce neuroinflammation and metabolic disturbances, the marked interspecies differences significantly limit the clinical translatability of these findings. Specifically: (1) fundamental divergence exists between murine and human immune responses—the relative contribution of TLR3/RLR signaling pathways in anti-RSV immunity differs, potentially leading to systematic underestimation of neuroinflammatory severity; (2) murine microglia exhibit oversimplified metabolic reprogramming (M1/M2 polarization) compared to humans, failing to recapitulate the complex metabolic heterogeneity in aging or neurodegenerative contexts; (3) methodologically, while human RSV typically invades the CNS via the respiratory-vagal axis, most murine studies employ direct intracranial inoculation, thereby omitting critical pathophysiological processes such as immune cell trans-barrier migration and remote cytokine cascades in the lung-brain axis.

The current scarcity of clinical data substantially impedes the validation of the hypothesis of energy metabolism dysfunction. Three major limitations persist: (1) large-scale epidemiological studies establishing direct links between RSV infection and neurodegenerative diseases are notably absent; (2) while retrospective analyses suggest potential cognitive decline in adulthood following childhood RSV infection, confounding factors (e.g., co-infections, socioeconomic disparities) remain incompletely adjusted for; (3) existing models fail to capture the profound pathological heterogeneity of human neurodegeneration, particularly gene-environment interactions involving major genetic risk factors (e.g., APOE ε4 genotype). Carriers exhibit markedly impaired mitophagy capacity that may exacerbate RSV-induced metabolic damage, yet such interactions are seldom incorporated in animal studies [74].

## 4. Therapeutic Implications and Future Directions

While current preclinical data point to a plausible link between RSV infection and metabolic disturbance in neural cells, several caveats should be noted: the extrapolation from animal models to human pathology remains speculative, the temporal relationship between RSV exposure and neurodegeneration onset has not been confirmed, and competing explanations cannot be ruled out. Alternative interpretations of the observed phenomena should be considered. For instance: (1) The metabolic alterations could represent epiphenomena of generalized neuroinflammation rather than RSV-specific effects. (2) Pre-existing subclinical neurodegeneration may increase susceptibility to RSV-induced damage. (3) Coinfections with other pathogens may confound the apparent RSV-neurodegeneration association.

Although the current treatment strategies targeting RSV-exacerbated neurodegeneration remain speculative, they have gained traction from preclinical data. Antioxidants (e.g., N-acetylcysteine) reduce ROS-mediated mitochondrial damage in RSV-infected neuron-microglia co-cultures, improving cell survival by 30–40% [75]. Metabolic regulators that enhance mitochondrial biogenesis may attenuate the RSV-induced glycolytic shift, reducing microglial M1 polarization and cytokine release [76]. Clinical trials evaluating these agents in RSV-associated encephalitis are warranted, particularly in high-risk populations (e.g., elderly and immune-compromised).

While energy metabolism dysfunction is a hallmark of RSV neurotoxicity, it remains unclear whether this is an RSV-specific mechanism or whether it is shared with other neurotropic respiratory viruses (e.g., influenza, SARS-CoV-2). Unlike RSV, the influenza virus primarily disrupts mitochondrial dynamics via M2 protein-mediated ion channel dysfunction, whereas SARS-CoV-2 targets angiotensin-converting enzyme 2 (ACE2) receptors, leading to distinct metabolic perturbations [79]. Clarifying RSV’s unique metabolic signatures of RSV, for example, NS1-mediated mitophagy vs. other viral apoptotic pathways, will refine therapeutic targeting.

The "energy metabolism disorder hypothesis" relies heavily on in vitro and murine data. Human postmortem brain tissue from RSV-infected individuals with neurodegenerative diseases is scarce, and longitudinal studies tracking RSV exposure to AD/PD incidence are lacking. Future research should integrate multi-omics approaches (metabolomics and proteomics) with clinical datasets to validate pathway specificity and identify diagnostic biomarkers (e.g., CSF lactate and mitochondrial DNA mutations).

## 5. Conclusion

RSV infection represents a novel risk factor for neurodegeneration, with energy metabolism dysfunction as the central pathological node. Addressing the limitations in clinical evidence, molecular details, and model translatability will accelerate the development of targeted interventions. By distinguishing RSV’s unique mechanisms of RSV from other viruses and prioritizing human-relevant studies, this hypothesis can evolve from preclinical observations to actionable therapeutic strategies.
